# Serratiopeptidase: a well-known metalloprotease with a new non-proteolytic activity against *S. aureus* biofilm

**DOI:** 10.1186/s12866-015-0548-8

**Published:** 2015-10-09

**Authors:** L. Selan, R. Papa, M. Tilotta, G. Vrenna, A. Carpentieri, A. Amoresano, P. Pucci, M. Artini

**Affiliations:** Department of Public Health and Infectious Diseases, Sapienza University, p.le Aldo Moro 5, 00185 Rome, Italy; Department of Chemical Sciences, Federico II University, Complesso Universitario Monte Sant’Angelo, Via Cinthia 4, 80126 Naples, Italy; CEINGE Advanced Biotechnology Scarl, Via Gaetano Salvatore 486, 80145 Naples, Italy

**Keywords:** Serratiopeptidase, Biofilm, S. aureus, Antivirulence

## Abstract

**Background:**

The use of indwelling medical devices is associated with a significant risk of infections by *Staphylococcus aureus* (*S. aureus*) which possesses a variety of virulence factors including many toxins and the ability to invade eukaryotic cells or to form biofilm on biotic and abiotic surfaces. The virulence factors above described are often related to proteins exposed on the bacterial surface. Blocking *S. aureus* colonization may reduce the incidence of invasive infectious diseases.

Previously reports evaluated the anti-infective properties of serratiopeptidase (Spep), an extracellular metalloprotease produced by *Serratia marcescens* ATCC 21074 (E-15), in impairing virulence-related staphylococcal properties, such as attachment to inert surfaces and adhesion/invasion on eukaryotic cells. However, to date its mechanism of action is unknown.

**Methods:**

Spep gene was PCR amplified and cloned into expression vector pET28b(+). The mutant EspepA was constructed from plasmid pET28b-Spep applying the one-step overlap extension PCR strategy. There sulting plasmids were costransformed in EcBL21(DE3) cells with the plasmid pRuW4inh1 harboring the Erwinia chrysanthemi secretion system.

Bacterial pellets and supernatants were collected and analyzed by SDS-PAGE and zymography. The unambiguous identification and a detailed structure characterization of both the wild type and the mutant Spep were obtained by mass spectrometric analyses.

The resultant supernatants sterilized by filtration were separately used to condition biofilm formation of S. aureus. Quantification was based on crystal violet method.

**Results:**

In this work we constructed Spep mutant by substituting the glutamic acid in the catalytic site with a residue of alanine. In this manner we were able to evaluate the anti-biofilm activity of Spep mutant in absence of proteolytic activity. As expected, this mutant did not display protease activity but it retained its anti-biofilm properties, suggesting that this action is independent by enzymatic activity.

**Conclusions:**

New knowledge obtained from data reported in this paper calls attention to a novel mechanism of action of Spep. This protein could be developed as a potential “antipathogenic agent” capable to impair the ability of *S. aureus* to form biofilm on prostheses, catheters and medical devices, exploiting a mechanism different from the proteolytic activity.

## Background

*S. aureus* is a flexible microbial pathogen frequently isolated from community-acquired and nosocomial infections [1]. The rapid emergence of hospital associated, antibiotic resistant *S. aureus* is now recognized as major epidemiological problem worldwide [2]. Moreover, the ability of *S. aureus* to adhere on both eukaryotic cells and abiotic surfaces via cell wall proteins and to form biofilm are important virulence factors in chronic infections associated with implanted biomaterials, which are particularly difficult to eradicate [3–5]. Hence, not surprisingly, the interest in the development of alternative anti-infective approaches for the prevention and treatment of staphylococcal infections has increased in recent years [6–9]. An innovative approach should target *S. aureus* major virulence factors without affecting bacterial viability.

With the aim of targeting some surface-related virulence features of staphylococci our first choice was to use a protease. In literature is reported the in vivo effect of the protease Esp secreted by *S. epidermidis* acting as an anti-biofilm and anti-colonisation agent against *S. aureus* cells living in the same ecological niche [10]. Previously we focused our attention on serratiopeptidase (Spep), an extracellular metalloprotease produced by the Gram-negative opportunistic pathogen *Serratia marcescens* [11].

Spep contains a zinc binding consensus HEXXHXXGXXH, where the three histidine are zinc ligands, and the glutamic acid is the catalytic base [12]. Spep is commonly used as an anti-inflammatory agent and it has been shown to modulate adhesin expression in some bacterial species and to enhance antibiotic efficacy towards biofilm-forming bacteria [13–16].

Previously the ability of Spep to impair some virulence properties of different *S. aureus* strains, like attachment to inert surfaces and adhesion/invasion of eukaryotic cells was evaluated. The treatment with Spep is able to hinder the attachment to abiotic surface and the invasion of human cells by *S. aureus* strains without affect bacterial viability. Spep was found to affect different surface proteins in *S. aureus* biofilm [9, 13, 16]. However, previous work does not explain the mechanism used by Spep to impair the *S. aureus* biofilm.

With the aim to understand the mechanism of Spep action to impair the adhesive properties of *S. aureus*, we expressed a recombinant form with a residue of alanine in substitution of glutamic acid, to delete its proteolytic activity. Surprisingly this mutant form retains the anti-adhesive behavior suggesting a novel mechanism of action or regulatory function to date unknown.

## Methods

### Construction of the expression vectors pET28b-Spep and pET28b-Spep-EmutA

Genomic DNA preparation from *S. marcescens* ATCC 21074 E-15 (purchased by ATCC collection) was carried out as reported by Alonso and co-workers [17].

The pET28b-Spep gene expression vector was constructed by cloning DNA fragment corresponding to the coding gene of Spep of *S. marcescens* E-15 (Table 1) into pET28b (+) vector. Spep gene was PCR amplified by using the primers SPEP fw EcoRI/NcoI and SPEP rv EcoRI reported in Table 1.

**Table 1:** Oligonucleotides and plasmids used in this work

Plasmid	Description	References
pET28b-Spep	pET28b (+) containing the Spep gene of *S. marcescens* E-15	This work
pET28b-Spep-EmutA	pET28b (+) containing the Spep-EmutA gene of *S. marcescens* E-15	This work
pRuW4inh1	Secretion system of *Erwinia chrysanthemi*	[20]
Oligonucleotides		
Spep fw EcoRI/NcoI	5’-GGTAATGAGTGGAATTCAACCCATGGAATCTAC-3’	
Spep rv EcoRI	5’-CATCACCGAATTCGTCACCATCATGC-3’	
Spep_EmutA fw	5’-CGTTTACCCATGCGATTGGCCATGC-3’	
Spep_EmutA rv	5’-GCATGGCCAATCGCATGGGTAAACG-3’	

The amplified fragments were digested with NcoI and EcoRI and cloned in pET28b (+) corresponding sites. The nucleotide sequence was checked to rule out the occurrence any mutation during synthesis.

The mutant EspepA was constructed from plasmid pET28b-Spep applying the one-step overlap extension PCR (OOE-PCR) strategy [18, 19]. Either the fw/rev EmutA primers, reported in Table 1, were designed to introduce a single-point mutation, thereby substituting the glutamic acid residue (Glu) with an alanine residue (Ala). The resulting construct was indicated as pET28b-Spep-EmutA.

The nucleotide sequence was checked to rule out the occurrence any mutation during synthesis.

### Expression of recombinant Spep and Spep EmutA

The resulting constructs were separately costransformed in *Ec*BL21(DE3) cells with the plasmid pRuW4inh1 harboring the *Erwinia chrysanthemi* secretion system (kindly provided by prof. Wandersman) [20].

The recombinant Spep and Spep-EmutA genes were separately expressed in *Ec*BL21(DE3) cells, as follows: fresh cultures were inoculated in LB medium containing 50 μg/mL of kanamycin and 34 μg/mL chloramphenicol. The recombinant cells were grown over night at 37 °C. Bacterial cells were subsequently diluted 1/100 and grown until the OD600 reached 0.5. At this time point 5 mM IPTG was added to each culture and bacterial pellets and supernatants were collected after 4 h by centrifugation at 16627 *g*.

### SDS-PAGE and Zymography

Supernatants were analyzed by SDS-PAGE and zymography. A commercial sample of Spep (Takeda, Osaka, Japan) was used as control (5 μg).

SDS-PAGE was carried out by standard methods with SDS-polyacrylamide separating gel (10 % acrylamide pH 8.8) and constant voltage (180 V) at room temperature. Following electrophoresis, proteins were stained with Coomassie brilliant blue (BIORAD).

Renaturing SDS-PAGE was performed as previously reported by Artini and co-workers with some modifications [15]. SDS polyacrylamide separating gel (10 % acrylamide pH 8.8) containing 0.5 % casein (wt/v) provided by Sigma was used to detect the lytic activity. After electrophoresis gels were soaked in distilled water at room temperature and then were transferred into the renaturing buffer (50 mM TrisHCl pH 8 containing 1 % Triton ×100) and shaken at 70 rpm for 2 h at 37 °C to allow renaturation.

The renatured proteins appeared as clear translucent bands on blue opaque background after Coomassie brilliant blue staining.

### In situ digestion and Mass Spectrometry analysis

The Coomassie blue stained protein bands corresponding to the wild type and mutant Spep were excised from the gel and in situ digested with trypsin. Protein bands were washed with acetonitrile (ACN) and then with 0.1 M ammonium bicarbonate. Protein samples were reduced by incubation with 10 mM DTT for 45 min at 56 °C. Cysteines were alkylated by treatment with 5 mM iodoacetamide for 15 min at room temperature in the dark. Gel particles were then washed with ammonium bicarbonate and ACN. Tryptic digestion was carried out using 12.5 ng/μl of enzyme in 50 mM ammonium bicarbonate pH 8.5 at 4 °C for 4 h.

The buffer solution was then removed and a new aliquot of enzyme/buffer solution was added for 18 h at 37 °C. A minimum reaction volume, enough for the complete rehydratation of the gel was used. Peptides were then extracted, washing the gel particles with 20 mM ammonium bicarbonate and 0.1 % TFA in 50 % ACN at room temperature and then lyophilized.

The resulting peptide mixtures were analyzed by LC/MS-MS (3520 Chip Q-Tof, Agilent) and the proteins were identified in the NCBInr database using an in house version of the MASCOT software.

### Microtiter plate biofilm assay

Quantification of in vitro biofilm production was based on the method described by Christensen with slight modifications [15]. Briefly, the wells of a sterile 48-well flat bottomed polystyrene plate were filled with an opportune volume of BHI and 5 % of cell free supernatants derived form bacterial cultures harboring pET28b-Spep and pET28b-Spep-EmutA, 10-fold concentrated with 10 kDa MWCO and sterilized by filtration. A suitable dilution of *S. aureus* 6538P culture in exponential growth phase (about 0.1 OD 600 nm) was added into each well. As control, biofilm formation was also performed using Spep purchased by Takeda at a final concentration of 200 U/mL (80 μg/mL).

The sterile 48-well flat-bottomed polystyrene plate was incubated for 24 h at 37 °C. Then, the supernatant cultures were removed and adhered cells were washed with double-distilled water and stained with 0.1 % crystal violet. After adhered cells were rinsed twice with double-distilled water, and thoroughly dried. The dye bound to adherent cells was solubilized with 20 % (*v/v*) acetone and 80 % (*v/v*) ethanol. The OD of each well was measured at 590 nm. Each data point is composed of four independent samples.

## Results and discussion

### Analysis of expression and enzymatic activity of recombinant Spep and Spep EmutA

The effect of SPEP treatment on virulence properties of different *S. aureus* strains, such as attachment to inert surfaces and adhesion/invasion of eukaryotic cells, was already investigated. Although SPEP did not affect bacterial viability, it is able to impair attachment to abiotic surface and invasiveness by *S. aureus* to human cells and affects both biofilm formation and biofilm dispersion. Nevertheless, previous work left unresolved by what mechanism Spep interferes with *S. aureus* biofilm. For this reason we decided to express a recombinant form of Spep.

Our recombinant form of Spep has a residue of alanine in substitution of glutamic acid in zinc binding consensus HEXXHXXGXXH. In this manner we were able to evaluate the anti-biofilm activity of the Spep mutant in absence of the proteolytic activity.

**Fig. 1:**
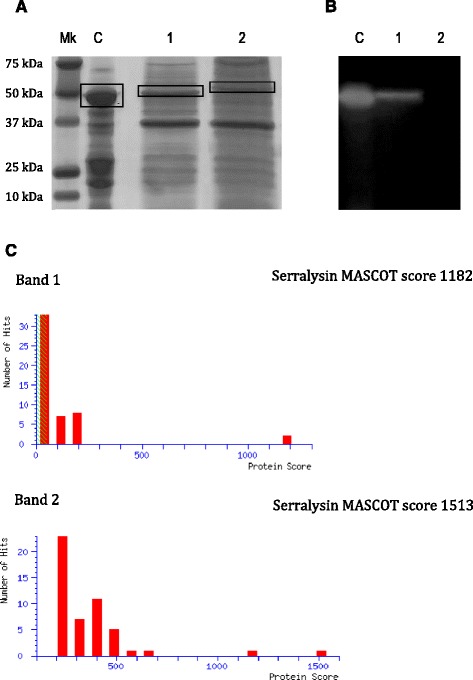
SDS-PAGE and zymogram analyses of supernatants of bacterial cultures harboring pET28b-Spep (*lane 1*) and pET28b-Spep-EmutA (*lane 2*), respectively. Panel **a** SDS-PAGE and Panel **b** zymogram assays. Mk: precision Plus prestained marker, Biorad (range 250–10 kDa); C: 5 μg of SPEP (2540 U mg^−1^, Osaka Japan). Panel **c** Mascot Search Results of the peptide mixtures derived from digestion with trypsin of the 1 and 2 bands performed with LC/MS-MS (3520 Chip Q-Tof, Agilent)

Recombinant forms of Spep and Spep-EmutA genes were separately expressed in *Ec*BL21(DE3) cells. Supernatants of recombinant bacterial cells were analyzed by SDS-PAGE and zymography (Fig. 1a-b). Results obtained demonstrated that soluble recombinant proteins were efficiently produced by *Ec*BL21(DE3) cells (Fig. 1a). Thus the presence of inclusion bodies in the cytoplasm was not evidenced (data not shown). Furthermore, recombinant proteins were correctly secreted in extracellular medium despite a small amount of them was present in the intracellular samples (data not shown).

The proteolytic activity of recombinant wild type Spep was confirmed by zymogram analysis using 0.5 % of casein as substrate (Fig. 1b). As clearly shown a translucent band is present in the supernatant deriving from the culture harboring pET28b-Spep (SN-Spep) while no protein band was detected in the lane corresponding to the supernatant deriving from the culture harboring pET28b-Spep-EmutA (SN-SpepEmutA).

### Identification and characterization of primary protein structure of recombinant spep and spep-emutA

The unambiguous identification and a detailed structure characterization of both the wild type and the mutant Spep were obtained by mass spectrometric analyses. The Coomassie blue stained protein bands corresponding to the wild type and mutant Spep (Fig. 1a, lanes 1 and 2 respectively) were excised from the gel and in situ digested with trypsin as reported in Materials and Methods section. The resulting peptide mixtures were analyzed by LC/MS-MS (3520 Chip Q-Tof, Agilent) and the proteins were identified in the NCBInr database using an in house version of the MASCOT software. The presence of Spep was confirmed in both bands showing a 62 % sequence coverage with 1182 MASCOT score for band 1 and 63 % sequence coverage with 1513 MASCOT score for band 2, respectively. Results are summarized in Fig. 1c.

A detailed description of the mutant Spep including the identification of the mutation site was obtained by MALDI mass spectrometric analysis of the tryptic peptide mixture generated by band 2. The accurate mass values recorded in the spectra were mapped onto the anticipated sequence of the Spep protein on the basis of their mass value and the trypsin specificity. The mass mapping strategy confirmed the identification and allowed us to verify most of the Spep primary structure. Moreover, the mass signal at m/z 3081.7 could only be assigned to the peptide 172–200 where a Glu residue at position 177 was substituted by Ala, thus confirming the occurrence of the expected mutant.

### Analysis of anti-biofilm activity of recombinant spep and spep emutA

**Fig. 2:**
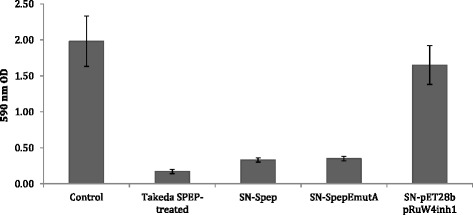
Biofilm formation of *S. aureus* 6538P in the presence of supernatant deriving from *E. coli* BL21DE3 harboring pET28b-Spep and pET28b-Spep-EmutA, respectively. Biofilm was reported as absorbance at 590 nm after crystal violet coloration. Results are representative of four independent experiments

Cell free supernatants derived form bacterial cultures harboring pET28b-Spep and pET28b-Spep-EmutA, 10-fold concentrated were separately used to condition biofilm formation of *S. aureus* 6538P. As control, biofilm formation was also performed using Spep purchased by Takeda (Osaka, Japan). Furthermore, biofilm formation was also performed in the presence of supernatant derived by *E. coli* BL21DE3, harboring both the vector pET28b and the plasmid pRuW4inh1. This control excludes the possibility that an *E. coli* component, secreted by the *Erwinia chrysanthemi* secretion system, could effect *S. aureus* biofilm formation. As already reported at a concentration of 200 U/mL (80 μg/mL) a quite complete disaggregation of biofilm was evidenced (less than 5 % of residual biofilm) [16]. Results obtained showed that supernatants containing wild type Spep and the inactivated mutant form of it were both effective to impair *S. aureus* bioflm formation (Fig. 2). Differently from other proteases like Esp [10], Spep inactivated mutant was still able to disaggregate biofilm. Conversely, Iwase and coworkers have identified that Esp mechanism of action in the biofilm reduction is dependent from its proteolytic activity [10]. Instead, by this data, we can confirm that Spep is able to induce the reduction of biofilm phenotype probably due to a regulating mechanism, not yet identified, independent from its proteolytic activity.

## Conclusions

Thinking about a new approach against the biofilm of *S. aureus*, a revolutionary strategy should target *S. aureus* major virulence factors without affecting bacterial viability. In this regard our results about the mechanism of action of Spep against *S. aureus* biofilm are really interesting. In literature is already reported the effect of the protease Esp secreted by *S. epidermidis* acting as an anti-biofilm and anti-colonisation agent against *S. aureus* cells living in the same ecological niche but, conversely to Esp*,* Spep does not get lose its activity when the catalytic site was disrupted [21]. New knowledge obtained from data reported in this paper, calls attention to a novel mechanism of action of Spep different from its proteolytic activity. In literature there are many cases of proteins known to have a specific biological role that however show alternative activities initially hidden [22]. Recently, several laboratories identifying proteins involved in the complex processes of replication, transcription and tumor suppression found that the ‘new’ proteins they discovered had another, previously identified, function [22]. A single protein with multiple functions might seem surprising, but there are actually many cases of proteins that ‘moonlight’, or have more than one role in an organism.

In this context, further efforts will be directed to the identification of the mechanism responsible for the biofilm regulation and to the identification of the molecular partners in *S. aureus* interacting with Spep.

## Abbreviations

Spep: Serratiopeptidase
ATCC: American Type Culture Collection
PCR (OOE-PCR): One-step overlap extension PCR
LB: Luria Broth
IPTG: Isopropil-β-D-1-tiogalattopiranoside
SDS-PAGE: Sodium Dodecyl Sulphate - PolyAcrylamide Gel Electrophoresis
ACN: Acetonitrile
DTT: Dithiothreitol
TFA: Trifluoroacetic acid
LC/MS-MS: Liquid chromatography–mass spectrometry tandem
MWCO: Molecular weight cut-off
SN: Supernatant.
